# Vitamin D Status and Acute Respiratory Infection: Cross Sectional Results from the United States National Health and Nutrition Examination Survey, 2001–2006

**DOI:** 10.3390/nu7031933

**Published:** 2015-03-13

**Authors:** Dominique J. Monlezun, Edward A. Bittner, Kenneth B. Christopher, Carlos A. Camargo, Sadeq A. Quraishi

**Affiliations:** 1 Tulane School of Public Health & Tropical Medicine, New Orleans, LA 70112, USA; E-Mail: dmonlezu@tulane.edu; 2 Tulane University School of Medicine, New Orleans, LA 70112, USA; 3 Harvard Medical School, Boston, MA 02115, USA; E-Mails: eabittner@bics.bwh.harvard.edu (E.A.B.); kbchristopher@partners.org (K.B.C.); ccamargo@partners.org (C.A.C.); 4 Massachusetts General Hospital, Boston, MA 02114, USA; 5 Brigham and Women’s Hospital, Boston, MA 02115, USA; 6 Harvard School of Public Health, Boston, MA 02115, USA

**Keywords:** vitamin D, 25OHD, respiratory, infection

## Abstract

Vitamin D is a promising, though under-explored, potential modifiable risk factor for acute respiratory infections (ARIs). We sought to investigate the association of vitamin D status with ARI in a large, nationally-representative sample of non-institutionalized individuals from the United States. We analyzed 14,108 individuals over 16 years of age in the National Health and Nutrition Survey (NHANES) 2001–2006 in this cross-sectional study. We used locally weighted scatterplot smoothing (LOWESS) to depict the relationship between increasing 25-hydroxyvitamin D (25OHD) levels and ARI. We then performed a multivariable regression analysis to investigate the association of 25OHD levels with ARI, while adjusting for known confounders. The median serum 25OHD level was 21 (IQR 15–27) ng/mL. Overall, 4.8% (95% CI: 4.5–5.2) of participants reported an ARI within 30 days before their participation in the national survey. LOWESS analysis revealed a near-linear relationship between vitamin D status and the cumulative frequency of ARI up to 25OHD levels around 30 ng/mL. After adjusting for season, demographic factors, and clinical data, 25OHD levels <30 ng/mL were associated with 58% higher odds of ARI (OR 1.58; 95% CI: 1.07–2.33) compared to levels ≥30 ng/mL. Among the 14,108 participants in NHANES 2001–2006, 25OHD levels were inversely associated with ARI. Carefully designed, randomized, controlled trials are warranted to determine the effect of optimizing vitamin D status on the risk of ARI.

## 1. Introduction

Acute respiratory infections (ARIs) are typically classified into two distinct categories—upper respiratory infections (URIs) and lower respiratory infections (LRIs). URIs involve the ear, nose, paranasal sinuses, pharynx, larynx, trachea, or bronchi, and are the most frequent reason for ambulatory medical visits in the United States [1]. On average, adults experience 2–4 URIs every year [2] and although mortality is relatively low (~3000 deaths/year) [3], the direct and indirect costs of non-influenza-related URIs exceed $40 billion, annually [4]. The financial impact of each influenza epidemic is estimated at $12 million in additional direct and indirect costs [5]. On the other hand, LRIs involve the alveoli and most commonly manifest as pneumonia [6]. In the United States, there are over 4 million ambulatory care visits for community-acquired pneumonia (CAP) annually [7], which lead to over 1 million hospitalizations [8] and roughly 50,000 deaths [9]. The direct and indirect costs attributable to CAP, in the United States alone, is in excess of $17 billion every year [10].

Given the significant impact of ARIs on health and healthcare expenditures, considerable interest has focused on factors that may influence susceptibility to such respiratory illnesses. Research over the past few decades has led to the appreciation of independent risk factors for ARIs, such as fall/winter season, age, exposure to cigarette smoke, population density, and various comorbidities (e.g., asthma and emphysema) [11,12,13,14]. Public health policies based on these risk factors, have at best, been modestly successful in limiting the annual incidence and spread of ARIs [14,15,16,17,18]. Since ARIs occur when microbes overrun natural defenses within various segments of the respiratory system, more recent efforts are investigating interventions that optimize host immune responses in at-risk individuals [19,20,21,22]. Since vitamin D is a potent regulator of the innate and adaptive immune systems [23], vitamin D status has been proposed as a potentially modifiable risk factor for ARIs [24]. To date, only a few, large studies have investigated the association of 25-hydroxyvitamin D (25OHD) levels (widely recognized as the best indicator of total body vitamin D status [25]) with either URIs or LRIs in the general population [26,27,28,29,30]. Since the source data in these studies were collected several decades ago, our goal was to investigate the association of 25OHD level with ARI in a more recent, large, nationally-representative sample of non-institutionalized individuals from the United States.

## 2. Materials and Methods

### 2.1. Source Data

The National Health and Nutrition Examination Survey (NHANES) data are nationally representative, cross-sectional samples of the non-institutionalized, civilian population of the United States [31]. They have been used extensively to report on the association of various biomarkers with major diseases. Conducted by the National Center for Health Statistics (Atlanta, GA, USA), and after three large phases between 1971 and 1994, the survey was converted into an annual process between 1999 and 2010. The most recent surveys with 25OHD assessments were 2001–2002, 2003–2004, and 2005–2006. During this period, 31,509 individuals were interviewed and 30,070 individuals completed physical examinations and laboratory testing. In addition, to allow for better population estimates, there was oversampling of individuals from the following categories: Age 12–19; age ≥70 years; non-Hispanic black; Mexican American; and low-income white. We conducted a secondary analysis of this large dataset (NHANES 2001–2006), after the Partners Human Research Committee (local Institutional Review Board) granted an “exempt” status for this retrospective study.

### 2.2. Data Collection

Detailed survey methods, including sampling, interview, examination, laboratory measurements, ethics approval, and informed consent have previously been reported [31]. In summary, the survey used a complex, stratified, multistage probability sample design to recruit nationally representative samples. Approximately 12,000 individuals were invited to participate in each 2-year NHANES cycle. The surveys were performed during scheduled in-home interviews to obtain demographic information as well as data on health and nutrition. Physical examinations and laboratory testing were performed in either a mobile examination center or during a home visit. Blood samples collected during the examination were centrifuged, aliquoted, and stored at −70 °C on-site. They were then shipped on dry ice to central laboratories, where they were stored at −70 °C until analysis. Serum 25OHD levels were measured using a radioimmunoassay kit after extraction with acetonitrile (DiaSorin, Stillwater, MN, USA) by the National Center for Environmental Health (Atlanta, GA, USA). Standards for 25OHD assays were released in July 2009 using isotope dilution tandem mass spectrometry (LC-MS/MS) candidate reference measurement procedures to assign certified values by the National Institute of Standards and Technology (NIST). The Centers for Disease Control and Prevention will release a revised analytic note in the future to improve 25OHD data interpretation including across NHANES 2001–2006 and NHANES III by generating regression equations to adjust 25OHD data using the NIST standards from LC-MS/MS. This will allow more accurate assessment of possible correlations with 25OHD and health conditions. NHANES also modified the 2003–2006 values for 25OHD in November 2010 to address observed drifts in assay performance following reagent and calibration lot fluctuation [32]. Quality control pool data from the drift period was used for a statistical adjustment model since the sample subject values would be independent of empirical trends [32].

### 2.3. Data Abstraction

We limited our analysis to the 14,108 individuals, 17 years and older, with reported 25OHD levels (primary exposure). To most accurately adjust for the effect of season on 25OHD levels, we recorded the 6-month block within which the laboratory samples were collected. We also abstracted information on all participants in the NHANES 2001–2006 datasets related to age, sex, race, body mass index (BMI), and poverty-to-income ratio (measure of socioeconomic status). We also abstracted smoking status, exposure to second-hand smoke, alcohol consumption, pneumococcal vaccination status, and several self-reported current diseases: Asthma, chronic obstructive pulmonary disease (COPD), congestive heart failure (CHF), diabetes mellitus (DM), and stroke. A diagnosis of COPD was based on responses to questions on emphysema and/or chronic bronchitis. Furthermore, we used laboratory data to determine the estimated glomerular filtration rate (eGFR) to assess for chronic kidney disease (CKD), and to document cases of neutropenia. The primary outcome, ARI, was based on the response to the question spanning both upper and lower ARI: “Did you have flu, pneumonia, or ear infections that started during [the past] 30 days?”

### 2.4. Statistical Analysis

All statistical analyses were performed using Stata 12.0 (StataCorp LP, College Station, TX, USA). Using survey commands, we applied the recommended subsample weights for the interview plus examination data to account for unequal probabilities of selection and to accurately represent estimates for the population of the United States. All of the results are presented as weighted values. We calculated variance based on NHANES-provided masked variance units using the Taylor series linearization method. All reported *p* values are 2-tailed, with *p* < 0.05 considered statistically significant. We calculated proportions with 95% confidence intervals (CIs) for demographic features and other factors thought to be related to ARI, overall and in the subset of participants with self-reported ARI, within 30 days of the interview.

Locally weighted scatter plot smoothing (LOWESS) was used to graphically represent the association between 25OHD level and the cumulative frequency of ARI. LOWESS is a type of nonparametric regression, which summarizes the relationship between two variables in a fashion that initially relies on limited assumptions about the form or strength of the relationship [33]. The rationale and methods underlying the use of LOWESS for depicting the local relationship between measurements of interest across parts of their ranges are available elsewhere [34].

For our primary analysis, we first considered serum 25OHD level as a continuous variable using LOWESS analysis, and then as a dichotomous variable based on the LOWESS results. To improve interpretability of the analysis, we converted some variables into commonly used groupings: Age (17–39, 40–59, and ≥60) and BMI in kg/m^2^ (<20, 20–24.9, 25–29.9, ≥30). In addition, we dichotomized other variables as follows: Season (1 May–31 October as high ambient ultraviolet B radiation *versus* 1 November–30 April as low ambient ultraviolet B radiation), race (non-white *versus* white), poverty-to-income ratio (≤federal poverty level *versus* >federal poverty level), alcohol consumption (≤30 drinks/month *versus* >30 drinks/month), CKD (eGFR <60 mL/min/1.73m^2^ *versus* ≥60 mL/min/1.73m^2^), and neutropenia (white blood cell count <3.5 × 10^9^/L *versus* ≥3.5 × 10^9^/L). We also dichotomized self-reported histories of: Active smoking, exposure to second-hand smoke in the household, pneumococcal vaccination, asthma, COPD, CHF, DM, and stroke. We determined unadjusted associations between risk factors and the outcome of ARI using the Pearson chi-squared test for categorical variables and simple ordinal logistic regression for ordinal variables. To evaluate the independent association between serum 25OHD level and ARI, we created multivariable models by progressively adding covariates that might confound or alter the association of 25OHD with ARI. All adjusted odds ratios (ORs) for the variables in the models are reported with 95% confidence intervals (CIs).

## 3. Results

Characteristics of the analytic sample are given in Table 1. The median age of the participants was 45 (IRQ 28–63) years; 51% were female and 51% were white. Overall, the median serum 25OHD level was 21 (IRQ 15–27) ng/mL. Overall, 4.8% (95% CI: 4.5–5.2) of the sample reported an ARI within 30 days before their NHANES interview. The proportion of participants with recent ARI, stratified by individual characteristics, is also presented in Table 1.

**Table 1:** Overall sample characteristics and sub-groups with acute respiratory infections.

Covariate	Total Number of Observations	Number Reporting Acute Respiratory Infection (Row %)	*p*-value
25-hydroxyvitamin D			
<10 ng/mL	1168	90 (7.7%)	<0.001
10–19.9 ng/mL	4958	262 (5.3%)	
20–29.9 ng/mL	5416	245 (4.5%)	
≥30 ng/mL	2566	82 (3.2%)	
Season			
High ambient ultraviolet B radiation	7468	236 (3.2%)	<0.001
Low ambient ultraviolet B radiation	6640	443 (6.7%)	
Age			
17–39 years	5934	285 (4.8%)	0.06
40–59 years	3836	208 (5.4%)	
≥60 years	4338	186 (4.3%)	
Sex			
Female	7252	386 (5.3%)	0.004
Male	6856	293 (4.3%)	
Race			
Non-white	7178	303 (4.2%)	0.001
White	6930	376 (5.4%)	
Poverty-to-income ratio			
≤Federal poverty limit	2408	148 (6.2%)	<0.001
>Federal poverty limit	10,361	454 (4.4%)	
Body mass index			
<20 kg/m^2^	744	34 (4.6%)	0.06
20–24.9 kg/m^2^	3787	160 (4.2%)	
25–29.9 kg/m^2^	4668	220 (4.7%)	
≥30 kg/m^2^	4390	241 (5.5%)	
Active smoker			
Yes	2801	173 (6.2%)	<0.001
No	3416	127 (3.7%)	
Second-hand smoke			
Yes	2767	176 (6.4%)	<0.001
No	11,227	494 (4.4%)	
Alcohol consumption			
≤30 drinks/month	952	37 (3.9%)	0.53
>30 drinks/month	7167	310 (4.3%)	
Pneumococcal vaccination			
Yes	867	35 (4.0%)	0.33
No	3278	158 (4.8%)	
Asthma			
Yes	1716	129 (7.5%)	<0.001
No	12,374	550 (4.4%)	
Chronic obstructive pulmonary disease			
Yes	929	87 (9.4%)	<0.001
No	11,694	504 (4.3%)	
Congestive heart failure			
Yes	421	36 (8.6%)	<0.001
No	12,916	556 (4.6%)	
Diabetes mellitus			
Yes	1290	72 (5.6%)	0.15
No	12,612	591 (4.7%)	
Chronic kidney disease			
eGFR < 60 mL/min/1.73m^2^	1697	82 (4.8%)	0.97
eGFR ≥ 60 mL/min/1.73m^2^	12,108	588 (4.9%)	
Stroke			
Yes	459	37 (8.1%)	<0.001
No	12,191	556 (4.6%)	
Neutropenia			
WBC ≥ 3.5 × 10^9^/L	13,958	672 (4.8%)	0.84
WBC < 3.5 × 10^9^/L	135	6 (4.4%)	

A two-tailed *p* < 0.05 was considered statistically significant, *p*-values are based on simple ordinal logistic regression (for ordinal variables) and chi-square test (for categorical variables).

**Figure 1:**
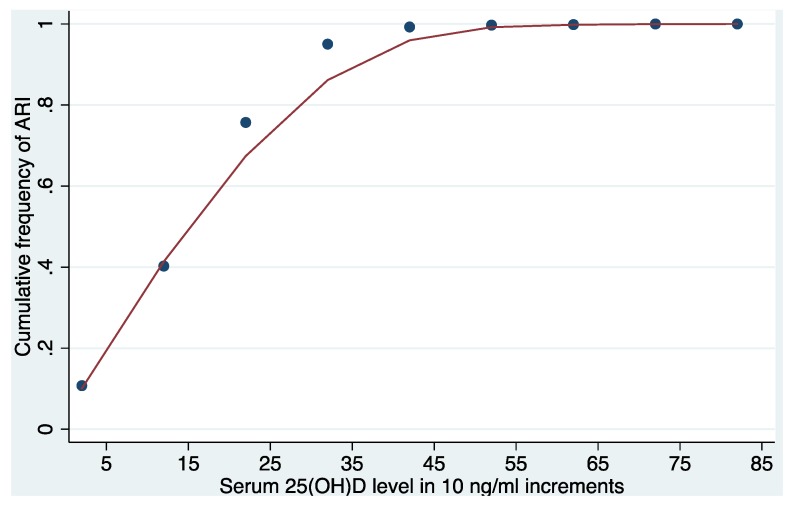
Near linear relationship of acute respiratory infection and 25-hydroxyvitamin D up to 30 ng/mL in LOWESS analysis. Locally weighted scatterplot smoothing analysis = LOWESS; 25OHD = 25-hydroxyvitamin D in 10 ng/mL increments; ARI = acute respiratory infection.

LOWESS analysis showed a near linear relationship between 25OHD level and the cumulative frequency of ARI up to 25OHD levels around 30 ng/mL (Figure 1). Between 25OHD levels of 30 ng/mL and 50 ng/mL there was an increasing flattening of the curve.

Based on the analysis, we selected a cut-point of 30 ng/mL in the model where 25OHD was considered as a dichotomous variable. Compared with individuals with 25OHD levels ≥30 ng/mL, those with levels <30 ng/mL had a 58% higher adjusted odds of ARI (OR 1.58; 95% CI: 1.07–2.33) within 30 days of the interview. Other independent risk factors associated with increased odds of ARI in this model were low ambient ultraviolet B radiation, active smoking, higher poverty-to-income ratio, history of COPD, and CHF (Table 2). Based on existing clinical guidelines [35], we further sub-categorized 25OHD levels <30 ng/mL as <10 ng/mL, 10 to 19.9 ng/mL, and 20 to 29.9 ng/mL. Looking within this large group of participants with 25OHD levels <30 ng/ml, we found higher adjusted odds of ARI for those with levels <10 ng/mL (OR 2.75; 95% CI: 1.67–4.54), 10–19.9 ng/mL (OR 1.36; 95% CI: 0.88–2.08), and 20–29.9 ng/mL (OR 1.55; 95% CI: 1.02–2.36), compared to individuals with levels of 30 ng/mL or higher.

**Table 2:** Multivariable model with odds ratios for acute respiratory infection risk factors.

Risk Factor	Odds Ratio (95% Confidence Interval), *p*-value
25OHD (<30 ng/mL *versus* ≥30 ng/mL)	1.58 (1.07–2.33), *p* = 0.023
Low ambient UVB radiation (1 November–30 April)	2.19 (1.69–2.85), *p* < 0.001
Poverty-to-income ratio (≤FPL *versus* >FPL)	1.47 (1.10–1.96), *p* = 0.009
Active smoking	1.39 (1.01–1.91), *p* = 0.046
Chronic obstructive pulmonary disease	1.94 (1.39–2.72), *p* < 0.001
Congestive heart failure	1.85 (1.15–2.97), *p* = 0.012

25OHD = 25-hydroxyvitamin D; UVB = ultraviolet B radiation; FPL = Federal poverty limit.

## 4. Discussion

In this large, nationally-representative sample of non-institutionalized individuals in the United States, we investigated whether 25OHD level was associated with ARI. We demonstrated that 25OHD levels were inversely associated with the odds of ARI. This relationship was most pronounced when comparing individuals with 25OHD levels <10 ng/mL to those with levels ≥30 ng/mL. While previous studies have shown that vitamin D status is associated with the risk of specific respiratory diseases [26,27,28,29,30], our work demonstrates a near linear relationship between vitamin D status and the cumulative frequency of ARIs over a commonly encountered range of 25OHD levels in the general population.  Such a growing body of evidence may thus support later randomized controlled trials to test the hypothesis that a therapeutic window of vitamin D supplementation may help reduce the risk of ARI. However, given the cross-sectional design of the present study, a causal inference about the effect of low 25OHD levels and higher risk of ARI is not possible. Nonetheless, the biological plausibility remains undeniable.

Cells of the innate and adaptive immune system express the vitamin D receptor [36], and in low vitamin D states, dysfunctional macrophage activity becomes evident [37]. Moreover, vitamin D is necessary for interferon-γ dependent T cell responses to infection [38] and it is also an important link between Toll Like Receptor (TLR) activation and antibacterial responses [39]. Macrophages stimulated by TLR-ligands induce further vitamin D receptor expression [40] and conversion of 25OHD to its most biologically active form (1,25-dihydroxyvitamin D) [41]. This, in turn, leads to increased production of cathelicidin, an endogenous antimicrobial peptide with potent activity against bacteria, viruses, fungi, and mycobacteria [42,43,44]. Cathelicidin is also highly expressed by epithelial cells at natural barrier sites (e.g., skin, gut, lungs) and may represent an important first-line of defense for the innate immune system [45] against pathogens that lead to ARI.

Although observational studies have suggested a link between vitamin D status and ARI [26,27,28], evidence from randomized controlled trials (RCTs) are conflicting. While one meta-analysis of existing RCTs concluded that there was no benefit to vitamin D supplementation for the prevention of ARIs [46], two separate meta-analyses, performed by independent research teams, found a lower risk of ARIs in vitamin D-treated groups [47,48]. Such discrepancies may be, at least partially, explained by the heterogeneity of the data included in the meta-analyses—e.g., the age of subjects (adults *versus* children), health status of participants (general population *versus* inpatients), frequency of vitamin D dosing (daily *versus* intermittent), dose of vitamin D supplementation, and baseline vitamin D status of the subjects. Nonetheless, these studies underscore the need for adequately powered, well-designed RCTs to determine whether vitamin D may be beneficial for the prevention of ARIs in the general population.

While our results provide further compelling evidence to suggest that vitamin D status may be a modifiable risk factor for ARIs in the general population, it is important to discuss the potential limitations of the present study. As with all observational studies, and any cross-sectional research design, there is potential for confounding due to the lack of a randomly distributed exposure. Selection bias may be present, since 25OHD was not recorded for all participants in the NHANES 2001–2006 survey. And despite adjusting for multiple potential confounders, there may still be residual confounding, which could account for the observed differences in outcomes. In particular, low 25OHD levels may be a reflection of poor general health or suboptimal nutritional state, for which we are unable to fully adjust. We are also unable to fully adjust for lack of sun exposure, use of sunscreens, physical activity, and influenza vaccination status. Given the confines of the NHANES survey, a further limitation is that we were unable to control for the exact amount of time between the self-reported ARI and the timing of blood draws (*i.e.*, the ARI may have occurred at any point up to 30 days before the survey). As such, we cannot rule out the possibility of reverse causation (*i.e.*, low 25OHD levels lead to ARIs *versus* ARIs lead to low 25OHD levels). However, in non-hospitalized individuals, 25OHD levels tend to be relatively consistent over time (intra-person Pearson correlation coefficient of 0.70 at three years between blood draws following adjustments for age, race, and season) [49]. Nonetheless, vitamin D status may be influenced by acute illness [25], and therefore 25OHD levels may have been different at the time that participants developed ARIs. Also, the NHANES dataset relies on a self-reported history of ARI, which may be prone to recall bias and is less valid as a measurement of true ARI status compared to evaluation by a medical professional. And finally, the ARI definition can include upper and lower respiratory tract infections that may be caused by different pathogens. These and other potential issues will need to be addressed in future studies in order to replicate and extend our findings.

## 5. Conclusions

In summary, these data demonstrate that low 25OHD levels are strongly associated with ARIs in a large, nationally-representative sample of non-institutionalized individuals from the United States. Longitudinal studies are required to confirm our findings and establish the mechanisms underlying these observations. Moreover, high-quality, randomized, controlled trials are warranted to determine whether vitamin D supplementation in individuals with low vitamin D status may affect the incidence and severity of ARIs in the general population.
